# *CDE6* Regulates Chloroplast Ultrastructure and Affects the Sensitivity of Rice to High Temperature

**DOI:** 10.3390/plants15020284

**Published:** 2026-01-17

**Authors:** Shihong Yang, Biluo Li, Pan Qi, Wuzhong Yin, Liang Xu, Siqi Liu, Chiyu Wang, Xiaoqing Yang, Xin Gu, Yungao Hu

**Affiliations:** 1 College of Life Sciences, Agriculture and Forestry, Southwest University of Science and Technology, Mianyang 621010, China; ysh15386631926@163.com (S.Y.); lbl13198089330@163.com (B.L.); qp13243@163.com (P.Q.); ywz191665587@126.com (W.Y.); xuliang@swust.edu.cn (L.X.); 17602835986@163.com (S.L.); varywang9@gmail.com (C.W.); 18090953003@163.com (X.Y.); guxin7052@163.com (X.G.); 2 Rice Research Institute, Southwest University of Science and Technology, Mianyang 621010, China; 3 State Key Laboratory of Crop Gene Resources and Breeding, Beijing 100081, China

**Keywords:** rice, chlorophyll biosynthesis, chloroplast development, photosynthesis, ribosome recycle factor, temperature sensitivity

## Abstract

Chloroplasts are key organelles in plants that carry out photosynthesis, convert light energy into chemical energy, and synthesize organic compounds. In this study, a stably heritable chlorophyll-deficient mutant was screened from the ethyl methanesulfonate-induced mutation library of Wuyunjing 21 (WYJ21). This mutant was designated as *chlorophyll deficient 6* (*cde6*). The *cde6* mutant exhibits a low chlorophyll content, photosynthetic defects, an impaired chloroplast structure, a significant reduction in the number of stacked thylakoid layers, and a yellow-green leaf phenotype in the early tillering stage. Through MutMap analysis, it was found that the *cde6* mutant harbors a single-base mutation (T→A) in the *LOC_Os07g38300* gene. This mutation results in an amino acid substitution from valine (Val) to aspartic acid (Asp) in the encoded protein, thereby affecting the protein’s structure and function. The mutation of *CDE6* leads to decreased expression of genes related to chloroplast development and chlorophyll biosynthesis. Further studies revealed that the *CDE6*, a potential chloroplast ribosome recycle factor, leads to high temperature sensitivity in rice when mutated. As high-temperature stress is a primary constraint to global rice productivity, the identification of *CDE6* provides a genetic target for improving thermotolerance. In conclusion, these findings demonstrate that *CDE6* plays a crucial role in chloroplast biogenesis and provide new insights into its regulatory function in high-temperature tolerance.

## 1. Introduction

Chloroplasts are organelles for photosynthesis in plants, which can convert light energy into chemical energy and plays a crucial role in plant growth and development [1]. Chlorophyll is synthesized within chloroplasts; it determines leaf color and influences seedling growth. Any defect in chloroplast development can lead to leaf color mutations, and a variety of such mutants have been identified, including pale green, yellow, white-striped, and albino mutants [2,3,4]. Changes in chloroplast structure usually affect the photosynthetic efficiency of mutants, leading to reduced crop yields and even plant death in severe cases. Additionally, in the plant resistance mechanism, chloroplasts also contribute to the heat stress response [5,6,7,8]. As a major abiotic stress, high temperature affects the yield and quality of crops in many regions around the world. Rice (*Oryza sativa* L.), a primary staple food for more than half of the global population, exhibits extreme sensitivity to heat stress, particularly during the reproductive and grain-filling stages. High-temperature exposure during these critical periods impairs pollen viability and reduces the seed-setting rate while increasing grain chalkiness, ultimately leading to substantial yield losses and significant deterioration in grain quality [9,10,11,12]. To date, numerous genes associated with high-temperature stress response and tolerance have been identified. Examples include genes that positively regulate high-temperature resistance, such as *TT1* [13], *TT2* [14], *TT3.1* [5] and *QT12* [15], as well as genes that negatively regulate high-temperature resistance, such as *TT3.2* [5] and *OsWRKY11* [16]. Therefore, mutants with defective chloroplast structure and development are becoming increasingly important in basic research and rice-breeding practices.

Now, numerous genes that influence chloroplast structure and development have been identified. For instance, in rice, *WLP3* encodes the chloroplast ribosomal small subunit protein L18; when a mutation occurs in *WLP3*, genes within the chloroplast fail to undergo normal translation, leading to an abnormal chloroplast structure [17]. *OsPUS1* mediates the pseudouridine (ψ) modification of chloroplast rRNAs in rice. *OsPUS1* accumulates substantially under low-temperature conditions, binds to chloroplast precursor rRNAs (pre-rRNAs), and catalyzes the pseudouridylation of rRNAs. Loss of *OsPUS1* function leads to a reduction in mature chloroplast rRNAs and pre-rRNAs, disrupts the activity and translation of chloroplast ribosomes, and results in abnormal chloroplast development and leaf albinism [18]. Mutation in the *YLWS* gene, which encodes a PPR protein, impairs the splicing of chloroplast genes under low-temperature conditions, thereby disrupting plastid ribosome assembly and protein translation, which in turn leads to aberrant chloroplast development and albino seedlings in rice [19]. Mutation of *OsPPR16* disrupts early chloroplast development in rice leaves, resulting in an albino phenotype at the seedling stage; however, the leaves gradually regain green pigmentation as the plants mature [20]. *AL5* encodes a chloroplast-localized P-type PPR protein. Its mutation disrupts thylakoid lamella formation and chloroplast development in early rice leaves, leading to a seedling albino phenotype, this phenotype gradually recovers after the three-leaf stage [21]. However, the albino phenotype of some mutants at the seedling stage does not recover in the later stages of their life cycle and even leads to plant death. For example, *OsCSL1* encodes an MKKK22 that is targeted to the endoplasmic reticulum (ER) and its mutation results in decreased expression of chloroplast-related genes, including chlorophyll biosynthesis genes, plastid-encoded RNA polymerases, nucleus-encoded RNA polymerases, and nucleus-encoded chloroplast genes [22].

In all living organisms, the genetic information carried by messenger RNA (mRNA) is decoded and translated into proteins by a highly conserved macromolecular machine—the ribosome. The translation cycle can be divided into four steps: initiation, elongation, termination, and recycling. In plants, ribosome recycling factors (RRFs) are essential for maintaining organellar proteostasis, which is fundamental to chloroplast development and photosynthetic efficiency. In *Arabidopsis thaliana*, *HFP108* encodes a chloroplast ribosome recycling factor, which influences chloroplast protein synthesis, photosynthesis establishment, and embryonic development processes [23]. A study by Rolland et al. demonstrated that the spinach ribosome recycling factor functions in chloroplasts [24]. However, in rice, there are no reports on the function of genes encoding ribosome recycling factors.

In this study, a stably heritable chlorophyll-deficient mutant was isolated and designated as *cde6*. This mutant exhibits a yellow-green leaf phenotype at the early tillering stage. *CDE6* encodes a ribosome recycling factor; its mutation leads to an impaired chloroplast structure and a significant reduction in the expression of genes associated with chloroplast biogenesis and chlorophyll biosynthesis. These results demonstrate that *CDE6* is essential for chloroplast development and plant growth in rice.

## 2. Results

### 2.1. Phenotypic Analysis of cde6 Mutant Rice

**Figure 1:**
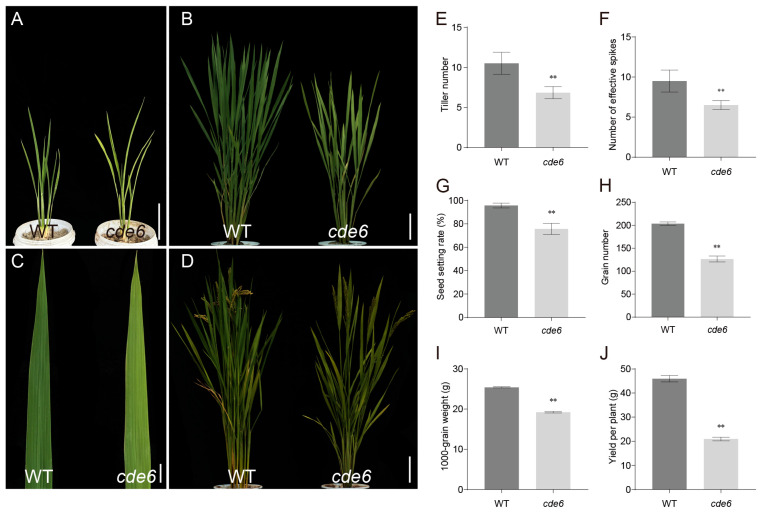
Phenotypic characteristics of the *cde6* mutant. (**A**) Early tillering stage phenotypes of WT and mutant *cde6*. Bar = 10 cm. (**B**) Peak tillering stage phenotypes of WT and mutant *cde6*. Bar = 10 cm. (**C**) Tillering stage leaf phenotypes. Bar = 1 cm. (**D**) Mature stage phenotypes of the WT and *cde6* mutant. Bar = 10 cm. (**E**–**J**) Comparison of tiller number, effective panicle number, seed setting rate, grain number per panicle, 1000-grain weight, and grain yield per plant. Values represent means ± SD (*n* = 10); (**: *p* < 0.01, ). (Student’s *t*-test).

To elucidate the molecular mechanisms governing chloroplast development, we isolated a stably inherited mutant from an EMS-mutagenized population of the WYJ21. Phenotypic characterization revealed that the *cde6* mutant exhibited a yellow-green leaf phenotype, starting from the early tillering stage (Figure 1A): a phenotype that is absent in wild-type (WT) plants at the corresponding developmental phase. The phenotype persisted when observed continuously until the mature stage (Figure 1B–D).

Agronomic trait analysis demonstrated that *cde6* mutants displayed significantly reduced tiller numbers and effective panicles compared to WT (Figure 1E,F). An evaluation of the panicle traits further showed that the mutant had a significantly lower seed-setting rate, grains per panicle, thousand-grain weight, and yield per plant, relative to WT (Figure 1G-J). These results collectively displayed that mutation of the *CDE6* locus not only impairs leaf color but also adversely affects key agronomic traits, including productive panicles, tiller number, grain weight, and plant productivity in rice.

### 2.2. Defects in Chloroplast Structure and Photosynthesis of cde6 Mutant

**Figure 2:**
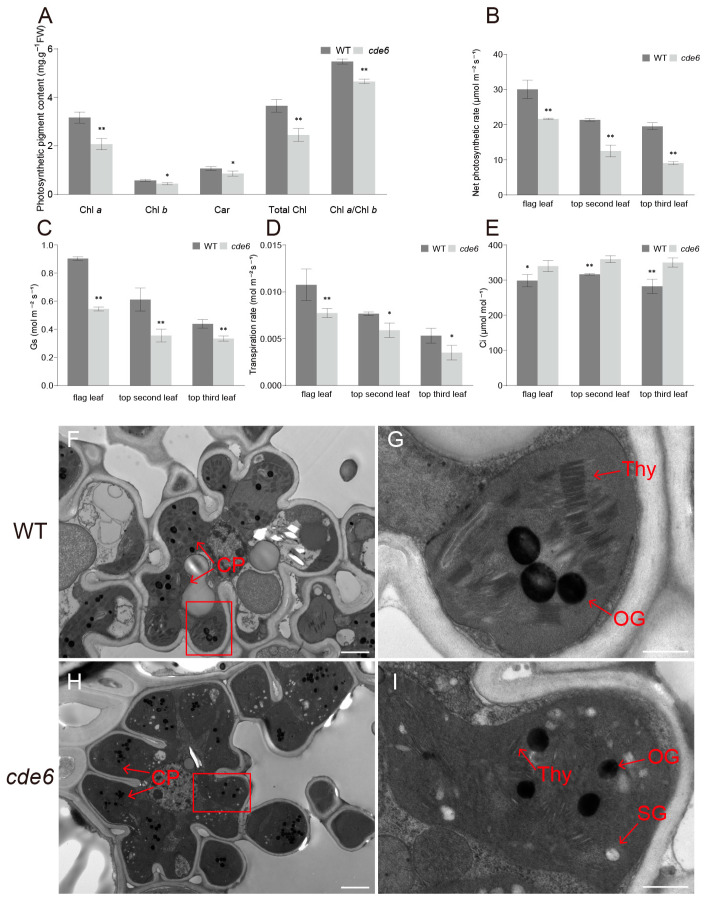
Photosynthetic traits and subcellular structure of *cde6* mutant. (**A**) Chlorophyll content in leaves of WT and *cde6* mutant plants at the heading stage. (**B**–**E**) Transpiration rate, net photosynthetic rate, stomatal conductance, and intercellular carbon dioxide concentration of WT and *cde6* mutant plants. Values represent means ± SD (*n* = 3); (*: *p* < 0.05, **: *p* < 0.01). (Student’s *t*-test). (**F**–**I**) TEM image of leaves on WT and *cde6*. OG, osmiophilic plastoglobuli; CP, chloroplast; SG, starch grain; and Thy, Thylakoids. (**F**,**H**) Scale bars = 2 µm. (**G**,**I**) Scale bars = 500 nm.

Changes in chlorophyll content usually induce alterations in plant leaf color phenotypes. To clarify the relationship between the yellow-green leaf phenotype of the *cde6* mutant and the chlorophyll content, we determined the pigment contents of WT and *cde6* mutant plants grown normally in the field. The results showed that the contents of chlorophyll *a*, chlorophyll *b*, carotenoids, total chlorophyll, and the ratio of Chl *a*/Chl *b* in the *cde6* mutant were significantly reduced (Figure 2A).

We further determined photosynthesis-related parameters in leaves from different positions of WT and *cde6* mutant plants at the full heading stage. The results indicated that the net photosynthetic rate of the flag leaf, the second leaf from the top, and the third leaf from the top of the *cde6* mutant reduced by 27%, 41%, and 53%, respectively, compared with that of WT (Figure 2B). Secondly, the stomatal conductance of the flag leaf, the second leaf from the top, and the third leaf from the top of the *cde6* mutant decreased by 39%, 41%, and 23%, respectively, relative to WT (Figure 2C). The transpiration rate of the *cde6* mutant reduced by 12%, 12%, and 19%, respectively, compared with WT (Figure 2D). In contrast, the intercellular CO_2_ concentration of the *cde6* mutant increased by 27%, 23%, and 32%, respectively, compared with WT (Figure 2E).

As the primary organelle for photosynthetic pigment biosynthesis and photosynthesis, the structural and functional integrity of chloroplasts directly affects leaf color phenotypes. TEM analysis showed that WT chloroplasts at the heading stage had an intact thylakoid membrane system, whereas the mutant exhibited significant structural abnormalities. Specifically, the mutant was characterized by scanty grana stacks, disordered lamellar orientation, and starch grain structures (Figure 2F–I and Figure S1). Collectively, these results demonstrate that the low photosynthetic pigment content, impaired photosynthetic function, and yellow-green leaf phenotype of the *cde6* mutant are caused by the disruption of chloroplast structural integrity.

### 2.3. Expression of Chloroplast Development Genes in WT and cde6 Mutant Plants

**Figure 3:**
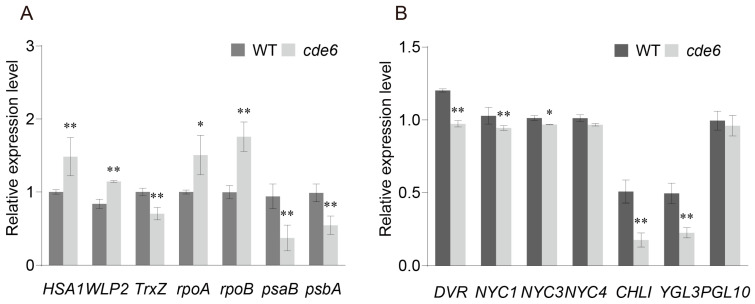
Relative expression of chloroplast-related genes in WT plants and the *cde6* mutant plants. (**A**) Relative expression of chloroplast-related genes in WT and *cde6* mutant at the heading stage. (**B**) Relative expression of chlorophyll biosynthesis-related genes in WT and *cde6* mutant at the heading stage. Values represent means ± SD (n = 3); (*: *p* < 0.05, **: *p* < 0.01). (Student’s *t*-test).

Given the impact of the *CDE6* mutation on chloroplast development and pigment biosynthesis, we further analyzed the expression levels of genes associated with early chloroplast development and chlorophyll biosynthesis in WT and *cde6* mutant plants at the heading stage. The results showed that compared with WT, the expression levels of multiple chloroplast development-related genes were significantly altered in the *cde6* mutant: the expression levels of *HSA1* (maintains chloroplast structural integrity), *WLP2* (regulates chloroplast biogenesis), *rpoA*, and *rpoB* (encodes chloroplast RNA polymerase subunits for plastid gene transcription) were significantly upregulated. In contrast, the expression of *psaB* (encodes PSI core subunit), *psbA* (encodes PSII core protein D1), and *TrxZ* (regulates chloroplast redox homeostasis) were significantly suppressed (Figure 3A).

In addition, compared with WT, the expression levels of chlorophyll biosynthesis-related genes—including *DVR* (divinyl chlorophyllide reductase), *NYC1* (chlorophyll *b* reductase gene), *OsNYC3* (encoding an α/β-hydrolase fold family protein), *NYC4* (reductase), *CHLI* (magnesium chelatase I subunit), *YGL3* (magnesium chelatase D subunit), and *PGL10* (protochlorophyllide oxidoreductase B) were all downregulated in the *cde6* mutant (Figure 3B). The suppressed expression of chlorophyll biosynthesis-related genes led to a reduction in chlorophyll content in plant leaves. Collectively, these results suggest that the abnormal expression of genes related to chloroplast development and chlorophyll biosynthesis may be associated with leaves turning yellow-green in the *cde6* mutant.

### 2.4. Accumulation of Reactive Oxygen Species in cde6

**Figure 4:**
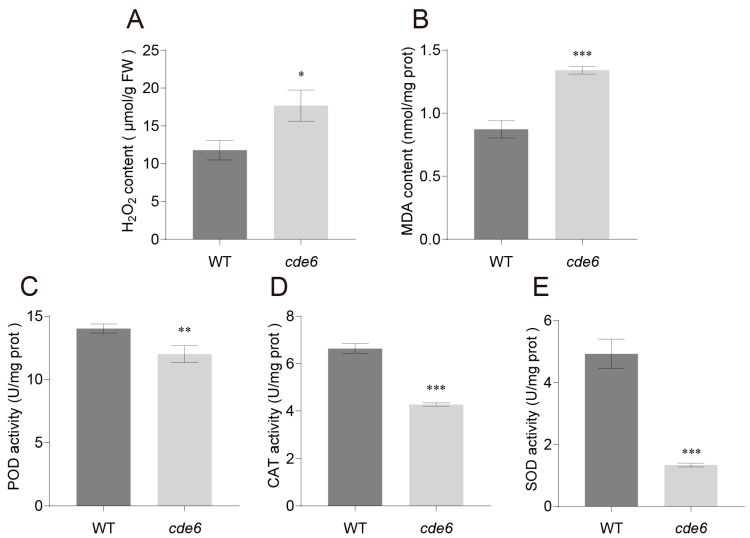
Determination of oxidative indices in WT and *cde6*. (**A**–**E**) The content of hydrogen peroxide (H_2_O_2_), malondialdehyde (MDA), activity of peroxidase (POD), catalase (CAT) and superoxide dismutase (SOD) in wild-type and *cde6* mutant leaves. Values represent means ± SD (*n* = 3); (*: *p* < 0.05, **: *p* < 0.01, ***: *p* < 0.001). (Student’s *t*-test).

Chloroplast development and chlorophyll biosynthesis are regulated by multiple factors. To investigate the accumulation of reactive oxygen species (ROS) in the *cde6* mutant at the tillering stage, we determined the physiological indicators related to the ROS metabolism. The results showed that compared with the WT, the contents of hydrogen peroxide (H_2_O_2_) and malondialdehyde (MDA) were significantly increased in the mutant (Figure 4A,B), while the activities of peroxidase (POD), catalase (CAT), and SOD (core enzymes of the plant ROS-scavenging system) were decreased (Figure 4C–E). These results indicate that the significant reduction in chlorophyll content and impaired chloroplast structure in the *cde6* may disrupt the balance between ROS production and scavenging, ultimately resulting in a certain degree of ROS burst in the mutant.

### 2.5. MutMap-Based Gene Mapping of CDE6

**Figure 5:**
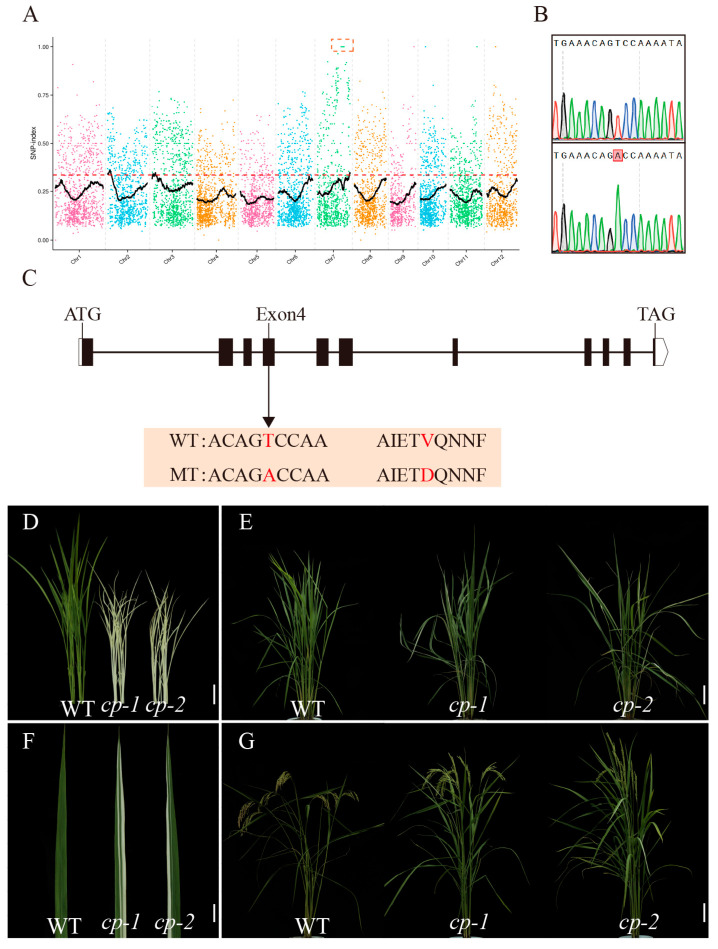
Molecular cloning of *CDE6*. (**A**) MutMap sequence localization of the *CDE6* locus to chromosome 7. (**B**) Sequencing revealed the presence of a single-base substitution in *cde6*. (**C**) Gene structure of the candidate gene *CDE6*. (**D**) Seedling-stage phenotypes of WT and knockout lines. (**E**) Tillering-stage phenotypes of WT and knockout lines. (**F**) Leaf phenotypes of WT and knockout lines at tillering stage. (**G**) Mature-stage phenotypes of WT and knockout lines. (**D**,**F**) Scale bars = 2 cm. (**E,G**) Scale bars = 10 cm.

To investigate the molecular mechanism underlying the yellow-green leaf phenotype of the *cde6* mutant, we developed an F_2_ population derived from the cross between WYJ21 and the *cde6* mutant. Phenotypic identification and genetic analysis of the F_2_ population showed that 443 plants exhibited the wild-type phenotype and 140 plants exhibited the yellow-green leaf phenotype of *cde6*, and the segregation ratio conformed to the Mendelian theoretical ratio of 3:1 (χ^2^ = 0.302 < χ^2^_0.05_ = 3.84) for single recessive nuclear gene inheritance. This confirmed that the mutant trait is controlled by a single nuclear gene (Supplementary Data S2). Thirty wild-type individuals and thirty F_2_ mutant individuals were selected to construct two separate DNA bulks, and MutMap-based gene mapping was performed, following whole-genome resequencing. SNP indices for each chromosome were obtained through MutMap analysis and a prominent peak in the SNP-index values was observed in the chromosome 7 region (Figure 5A). Subsequent sequencing of genes on chromosome 7 and analysis of mutation sites showed that only the mutation in the *LOC_Os07g38300* gene resulted in functional changes. A single-base substitution (T→A) was identified in this candidate gene. This mutation resulted in an amino acid substitution from valine (Val) to aspartic acid (Asp) in the encoded protein, thereby affecting the function of the protein (Figure 5B,C and Figure S2).

Furthermore, through co-segregation analysis, the mutant regions of 90 mutant individuals were amplified, and the results showed that all their genotypes were homozygous mutations, which further confirms the co-segregation of the point mutation with the yellow-green leaf phenotype (Figure S3). To verify whether *LOC_Os07g38300* corresponds to the *CDE6* gene, a knockout experiment was conducted on *LOC_Os07g38300*, and two independent homozygous mutants (*cp-1* and *cp-2*) were successfully obtained. These mutants exhibited a more severe albino phenotype at the seedling stage compared to the *cde6* mutant (Figure 5D). This albino phenotype was caused by a 1-base insertion and a 1-base deletion in the seventh exon, respectively, leading to frameshift mutations and premature termination of the translation (Figure S4). Notably, the albino phenotype gradually recovered after the three-leaf stage (Figure 5E–G). Collectively, these results demonstrate that mutations in *CDE6* are responsible for the impairment of chloroplast structure in rice.

### 2.6. Expression Pattern Analysis and Subcellular Localization of CDE6

**Figure 6:**
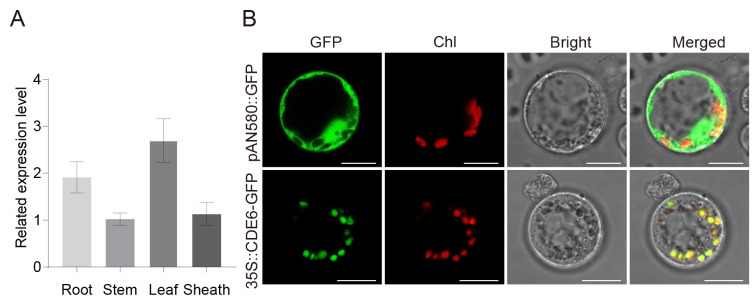
*CDE6* expression pattern analysis. (**A**) *CDE6* transcription level in various organs. (**B**) Subcellular localization of the pAN580-CDE6 in rice protoplasts. Fluorescence of GFP and chlorophyll autofluorescence (Chl) were detected by a confocal laser scanning microscope. Overlays of GFP and Chl image (Merged) were shown in right panels. Scale bars represent 10 µm.

The expression pattern of the *CDE6* gene was predicted using the RiceXPro database (https://ricexpro.dna.affrc.go.jp/ (accessed on 14 September 2025)), which revealed that *CDE6* exhibits a constitutive expression pattern across the different developmental stages of rice (Figure S5). Notably, its expression level is highest in leaves. To further verify the expression profile of *CDE6* in various tissues, roots, stems, leaves, and sheaths were collected from WYJ21, and quantitative real-time PCR (qRT-PCR) was performed to analyze the expression pattern of *CDE6*. The qRT-PCR results demonstrated that *CDE6* is expressed in roots, stems, leaves, and sheaths, with the highest relative expression level being in leaf tissues (Figure 6A).

Subcellular localization prediction using the Plant-mPLoc tool (http://www.csbio.sjtu.edu.cn/bioinf/plant-multi/ (accessed on 15 September 2025)) indicated that the CDE6 protein is targeted to chloroplasts. To validate the accuracy of this prediction, a pAN580 vector containing a 35S promoter-driven CDE6-GFP fusion protein expression system was constructed. The coding region of *CDE6* (with the stop codon removed) was fused with GFP to generate a transient expression vector. The GFP signal overlaps with the autofluorescence signal of chlorophyll, indicating that *CDE6* is primarily localized in chloroplasts (Figure 6B).

### 2.7. The cde6 Mutant Is Sensitive to High Temperatures

**Figure 7:**
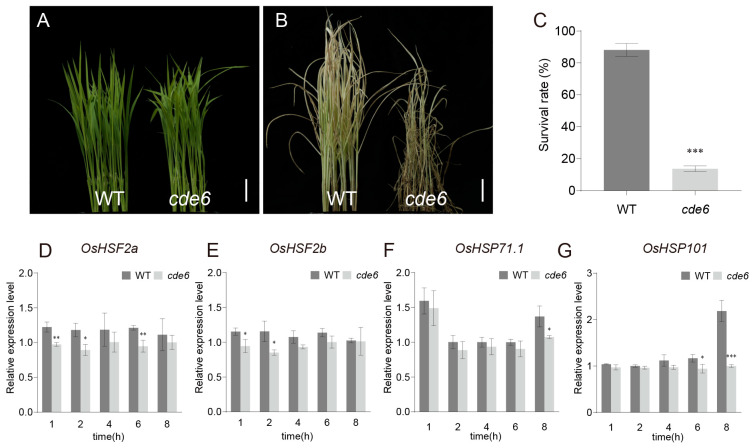
Response of the *cde6* mutant to high temperature. (**A**) WT and *cde6* mutant seedlings before high-temperature treatment. (**B**) Phenotypes of WT and *cde6* mutant seedlings after high-temperature treatment and 16 days of recovery. (**A**,**B**) Scale bars = 2 cm. (**C**) Survival rate statistics of WT and *cde6* mutant seedlings after high-temperature treatment. (**D**–**G**) Relative expression levels of HSFs and HSPs-related genes in seedlings of WT and *cde6* mutant under high-temperature stress. Values represent means ± SD (n = 3); (*: *p* < 0.05, **: *p* < 0.01, ***: *p* < 0.001). (Student’s *t*-test).

In previous reports, many chlorophyll-deficient mutants have been shown to be temperature-sensitive [4,25,26,27]. To determine whether *cde6* is sensitive to high temperatures, we subjected the WT and the *cde6* mutant to a high-temperature treatment at 42 °C for 25 h, followed by a recovery period at 28 °C for 16 days, after which the survival rate was calculated. The results are shown in the figures: after the high-temperature treatment, *cde6* seedlings exhibited varying degrees of leaf wilting and curling (Figure 7A,B). Following the recovery treatment, the survival rate of *cde6* was 14%, which was significantly lower than the 88% survival rate of WT (Figure 7C). In the process of plants responding to high-temperature stress, heat shock factors (HSFs) are a crucial class of transcription factors that regulate the expression of genes encoding heat-responsive proteins such as heat shock proteins (HSPs). This regulation initiates a series of complex physiological and biochemical processes to alleviate the damage caused to plants by high temperatures. Therefore, RT-qPCR was used to detect the expression levels of *OsHSFA2a*, *OsHSFA2b*, *OsHSP71.1*, and *HSP101* in *cde6* and WT seedlings at the seedling stage under high-temperature stress. The results showed that the expression levels of *OsHSFA2a*, *OsHSFA2b*, *OsHSP71.1*, and *HSP101* in *cde6* were lower than those in WT at 1 h, 2 h, 4 h, and 8 h after high-temperature treatment (Figure 7D–G). These results suggest that *CDE6* may affect the tolerance of rice seedlings to high-temperature stress by influencing the transcriptional levels of *OsHSFs* and *OsHSPs*.

## 3. Discussion

When chloroplast development and chlorophyll biosynthesis are disrupted, plants typically exhibit a variety of leaf color phenotypes [28], such as pale green, yellow, zebra stripe, and albino [29,30,31]. In this study, a stably heritable mutant was successfully identified via EMS-induced chemical mutagenesis. Its phenotypic characteristics emerged at the early tillering stage and persisted until the maturity stage (Figure 1A,D). At the heading stage, the contents of photosynthetic pigments (including chlorophyll *a*, chlorophyll *b*, and carotenoids) in the flag leaves of the *cde6* mutant showed extremely significant differences compared with the wild-type control (Figure 2A). This result confirms that the mutant has a specific genetic defect in the chlorophyll metabolic pathway, which impairs normal photosynthesis. Notably, the chlorophyll *b* content of this mutant significantly reduced, accompanied by a remarkable decrease in the chlorophyll *a*/chlorophyll *b* ratio. This alteration indicates that the light-harvesting antenna complexes in the *cde6* mutant are defective in assembly. The light-harvesting antenna complexes are mainly composed of chlorophyll *a*/*b*-binding proteins, and chlorophyll *b* is essential for the correct folding and stabilization of these proteins. Furthermore, the core function of chlorophyll *b* is to capture light energy and transfer it to the reaction center. Therefore, the impeded assembly of this complex will not only impair the stability of the photosynthetic apparatus but also reduce the efficiency of light energy utilization [32,33]. Chloroplasts possess an intrinsic ROS-scavenging system that protects them against oxidative damage [34,35]. In our study, the impaired chloroplast structure in the *cde6* mutant compromised chloroplast functions, consequently leading to elevated ROS levels. The impaired chloroplast structure affects the photosynthetic rate of rice leaves, thereby influencing the rice yield [36], such as the yellow-green leaf mutant *yl1*, which exhibits a severe loss of thylakoid lamellar structure and reduced yield [30]. Ultrastructural observations revealed that the thylakoid lamellae in ygl16 chloroplasts exhibited irregular distribution, with grana and stromal lamellae showing varying degrees of blurring and loosening, resulting in a disordered chloroplast structure. Consistently, the ygl16 mutant displayed a significantly lower net photosynthetic rate and stomatal conductance, accompanied by a higher intercellular CO_2_ concentration [37]. In this study, we compared the chloroplast structures of *cde6* mutant and WT plants and observed the severe impairment of chloroplast structure (Figure 2F–I). Additionally, we determined photosynthesis-related parameters: the net photosynthetic rate, stomatal conductance, and transpiration rate of the *cde6* mutant were all significantly lower than those of the WT. These photosynthetic defects further contribute to the reduced yield of the *cde6* mutant (Figure 2B–E).

To investigate the function of this gene, genetic analysis using an F_2_ population and MutMap sequencing revealed a single-base mutation (T→A) at the 32nd base of the fourth exon in *LOC_Os07g38300* on chromosome 7. This mutation causes a non-conservative amino acid substitution (Val→Asp), which is inferred to potentially trigger abnormalities in downstream physiological processes, leading to an impaired chloroplast structure and chlorophyll deficiency, thereby affecting the leaf color (Figure 5C). Knockout of *LOC_Os07g38300* in wild-type rice using CRISPR/Cas9 technology showed that the knockout plants exhibited a more severe albino phenotype at the seedling stage compared to the *cde6* mutant (Figure 5D). Specifically, the *cp-1* and *cp-2* lines had a 1-base insertion and a 1-base deletion in the 7th exon, respectively, both of which resulted in premature termination of translation—this explains why their phenotype was more severe than that caused by the single-base substitution in *cde6* (Figure S4). For instance, the *YGL3* gene is one of the key genes in the MEP pathway, encoding 4-hydroxy-3-methylbut-2-enyl diphosphate reductase localized on the thylakoid membrane. A single-base mutation in the *ygl3* mutant leads to an abnormal chloroplast ultrastructure and reduced chlorophyll content, resulting in yellowish-green leaves. In contrast, knockout of *YGL3* causes a frameshift mutation, which induces albinism at the seedling stage and death at the third-leaf stage [38]. As a temperature-sensitive mutant, *cde4* exhibits an albino phenotype at the seedling stage when grown at 20 °C. Under natural high-temperature conditions in the field, there are no significant phenotypic differences between *cde4* mutants and wild-type (WT) plants at the tillering and maturity stages. However, the knockout mutant of *CDE4* displays a more severe albino phenotype than the *cde4* mutant at both 20 °C and natural conditions. This extreme phenotype is caused by the insertion of one nucleotide into the second exon, which results in a frameshift mutation and premature termination of the translation [39].

*LOC_Os07g38300* is predicted to encode a ribosome recycling factor (RRF) in protein synthesis. To date, the function of genes encoding ribosome recycling factors (RRFs) has not been reported in rice. The amino acid sequence of *CDE6* shares 71.6% homology with the chloroplast-localized RRF of *Arabidopsis thaliana* (Figure S6). This chloroplast RRF in *Arabidopsis* is involved in protein synthesis, and its inactivation impairs chloroplast development and embryogenesis. In the *hfp108-1* mutant [23], the transcriptional levels of *psbA*/*psbB* and *psaA*/*psaB* remain at 50% of those in the wild type, while the protein levels of PsaA/PsaB drop to less than 5%. This confirms that the final accumulation of chloroplast-encoded proteins PsaA/PsaB is significantly regulated at the translational level. In the present study, the *cde6* mutant exhibits significantly downregulated expression levels of *psaB* and *psbA* (Figure 3A). We speculate that the impaired chloroplast ultrastructure in the *cde6* mutant, combined with the altered expression of genes involved in chloroplast development, collaboratively lead to the observed defects in chloroplast biogenesis. The normal function of chloroplasts relies on the coordinated expression of genes transcribed by nuclear-encoded RNA polymerase (NEP) and plastid-encoded RNA polymerase (PEP). Functional defects in the PEP complex within chloroplasts result in the upregulation of NEP-dependent genes and the downregulation of PEP-dependent genes [20,39]. For example, *CDE4* is a PEP-associated protein in rice. In the *cde4* mutant, the transcriptional levels of PEP-dependent genes (e.g., *psaA*, *psbA*, *psbB*, and *rbcL*) are significantly downregulated, whereas the expression levels of NEP-associated genes (e.g., *RpoTp*, *rpoA*, and *rpoB*) are significantly upregulated under low-temperature conditions [39]. Here, we observed that in the *cde6* mutant, the transcriptional levels of PEP-dependent genes (e.g., *psaB* and *psbA*) were significantly downregulated, while the expression levels of NEP-associated genes (e.g., *rpoA* and *rpoB*) were significantly upregulated. This result suggests that *CDE6* is also likely a gene involved in regulating chloroplast gene transcription in rice, and its function may be similar to that of *CDE4*, with a regulatory role in the transcriptional levels of PEP-dependent genes and NEP-related genes. OsTRXz interacts with WLP2 and HSA1/OsFLN2 to regulate the transcription of PEP-related genes and chloroplast biogenesis [40,41,42]. Loss of *TRXz* function leads to the disassembly of the PEP complex, directly inhibiting the transcription of key chloroplast genome-encoded genes (e.g., *psaA* and *psbA*) [43]. In this study, the expression level of *TRXz* was downregulated, whereas the expression levels of *HSA1* and *WLP2* were upregulated (Figure 3A). We hypothesize that this upregulation of *HSA1* and *WLP2* is a direct compensatory response to *TRXz* deficiency.

Under heat stress, plants rely on HSFs transcription factors to regulate the expression of HSPs and other heat-responsive protein genes, alleviating heat damage through complex mechanisms [44,45,46]. Under high-temperature conditions, HSPs function as molecular chaperones in response to heat stress. They prevent protein denaturation, maintain protein homeostasis, and thereby alleviate heat-induced damage to plants [47]. In the present study, the expression levels of the detected HSF and HSP genes in WT were higher than those in the *cde6* seedlings (Figure 7D–G). We propose that one of the potential causes for the altered thermotolerance in the mutant is that *CDE6* reduces the expression levels of *OsHSPs* by regulating the expression of *OsHSFA2s*.

## 4. Materials and Methods

### 4.1. Plant Materials and Growing Conditions

The *cde6* mutant was generated via ethyl methanesulfonate (EMS) mutagenesis, using WYJ21, a japonica rice variety, as the wild-type background. Through successive generations of self-pollination, a stable and heritable *cde6* mutant line was obtained. All rice plants were grown under natural field conditions at the Southwest University of Science and Technology, with ambient temperatures ranging from 28 °C to 37 °C. Field management practices followed standard agricultural protocols. Rice seedlings were cultured in a growth chamber under the following conditions: 28°C (16 h light)/25°C (8 h dark), with a light intensity of 8000 lx (light period) and 0 lx (dark period). 

### 4.2. Agronomic Trait Evaluation

Both WT and *cde6* mutant rice plants were grown in the field, with 6 rows per genotype and 10 plants per row. At the maturity stage, 10 plants were randomly selected from the middle of each row for agronomic trait evaluation. Student’s t-test was used for statistical analysis.

### 4.3. Measurement of Chlorophyll Content

The determination of leaf chlorophyll content was performed according to the previous method [48]. At the heading stage, the photosynthetic pigment content in leaves of WYJ21 and the *cde6* mutant was determined. Flag leaves from the plants were selected; surface moisture was wiped off, main leaf veins were removed, and approximately 0.05 g of leaf tissue from the same region was weighed. The weighed leaf tissue was cut into small pieces and placed into a 50 mL centrifuge tube, followed by the addition of 25 mL of extraction solution (acetone/ethanol = 1:1, *v*/*v*) that had been pre-cooled in a 4 °C refrigerator. The mixture was subjected to decolorization in the dark for 1–2 days, with occasional shaking during this period to ensure sufficient mixing between the leaf tissue and the extraction solution. Using the acetone/ethanol (1:1, *v*/*v*) mixture as the reference solution, 1–1.5 mL of the sample extraction solution was transferred into a cuvette. The absorbance values of WT and *cde6* mutant samples were measured separately at wavelengths of 470 nm, 645 nm, and 663 nm, and the chlorophyll content was calculated based on these values. Each sample was analyzed with three biological replicates.

### 4.4. Transmission Electron Microscopy (TEM) Observation

At the tillering stage, leaf segments (approximately 1 cm in length) were excised from WT and *cde6* plants and fixed in a 3% glutaraldehyde solution. After vacuum infiltration to ensure complete submersion of the samples, the samples were stored at 4 °C. Subsequent sample processing was performed by Lilai Biomedical Co., Ltd. (Chengdu, China). TEM observations were conducted using a JEM-1400FLASH transmission electron microscope (JEOL Japan Electronics Co., Ltd., Tokyo, Japan).

### 4.5. Determination of Physiological Indices

Leaves of WT and the *cde6* mutant were collected; after removing the main leaf veins, 0.1 g of fragmented leaf tissue was weighed and placed into a pre-chilled mortar. A 4 °C pre-cooled 0.1 M phosphate-buffered saline (PBS) solution was added at a weight-to-volume ratio (*w*/*v*) of 1:9, followed by the addition of an appropriate amount of quartz sand. The mixture was ground into a homogenate on ice. Low-temperature centrifugation (4 °C, 3500 rpm for 10 min) was performed to separate the supernatant. Commercial assay kits from the Nanjing Jiancheng Bioengineering Institute (Nanjing, China) were used to determine and analyze the contents of catalase (CAT), malondialdehyde (MDA), and superoxide dismutase (SOD) at wavelengths of 405 nm, 532 nm, and 550 nm, respectively. Each test sample was subjected to three biological replicates, and the resulting data were statistically analyzed using Student’s *t*-test.

### 4.6. Gene Mapping

Following phenotypic analysis of an F_2_ population from cross WYJ21 × *cde6* mutant, selected leaves were chosen to construct WT and mutant pools from which DNA was extracted and sequenced. The candidate gene was identified by a MutMap approach, based on alignment to the WT WYJ21 genome [49].

### 4.7. Subcellular Localization

The coding region of *CDE6* was amplified from the WYJ21 genome. Using pAN580 as the backbone vector, the *Xba*I and *Bam*HI restriction enzyme sites were selected to construct the relevant expression vector. The successfully constructed recombinant vector was designated as 35S::CDE6-GFP, with pAN580 serving as the empty vector control. This recombinant vector and the control vector (GFP alone) were separately transformed into rice protoplasts, followed by transient expression assays performed according to the previous method [50]. Green fluorescent protein (GFP) signals and chloroplast autofluorescence signals were observed and imaged using a Leica M205c confocal laser scanning microscope.

### 4.8. CRISPR/Cas9 Knockout of cde6

To generate pCRISPR-*CDE6* gene-editing knockout plants harboring dual targets, single-guide RNAs (sgRNAs) were designed using E-CRISP (https://www.e-crisp.org/, (accessed on 15 March 2024)). Two target sequences were cloned into the sgRNA expression cassettes of vectors pYLgRNA-OsU6a and pYLgRNA-OsU6b via overlap extension PCR, yielding the fragments pU6a-T1-sgRNA and pU6a-T2-sgRNA, respectively. These two fragments were then ligated by an overlap extension PCR, and the resulting fusion fragment was inserted into the *Apa*I and *Pst*I restriction sites of the pYLCRISPR-Cas9Pubi-H vector through homologous recombination to generate the final plasmid. Subsequently, this construct was transformed into Zhonghua11 via an Agrobacterium-mediated method. All primers used for vector construction and verification are listed in (Supplementary Data S1).

### 4.9. Quantitative Real-Time PCR

Total RNA was extracted from leaves and reverse-transcribed into cDNA using the Pure Plant RNA Preparation Kit (TIANGEN, Beijing, China). The rice actin gene was used as the internal reference. Quantitative real-time PCR (RT-qPCR) assays were performed using the SYBR Green Pro Taq HS Premix qPCR Kit (Accurate Biology, Hunan, China). All RT-qPCR primers are listed in (Supplementary Data S1).

### 4.10. Seedling-Stage High-Temperature Treatment

Approximately 150 seeds each of WYJ21 and the mutant were soaked in a greenhouse at 28 °C. After the seeds turned white and were germinated, they were individually placed in PCR plates and grown normally in an artificial climate incubator at 28 °C for 14 days. Subsequently, the seedlings were subjected to heat treatment at 42 °C for 25 h, followed by a 16-day recovery culture at 28 °C, and the survival rate was calculated thereafter.

## Data Availability

The datasets supporting the conclusions of this article are included within the article and its Supplementary Files.

## Supplementary Materials

The following supporting information can be downloaded at: https://www.mdpi.com/article/10.3390/plants15020284/s1, Supplementary Data S1: All primer sequences used in this study; Supplementary Data S2. Segregation ratio of hybrid progeny; Figure S1. Chloroplast ultrastructures of wild-type and *cde6* mutant rice. Figure S2. Amino acid sequences’ alignment between WT and *cde6*; Figure S3. Co-segregation verification of genotypes and phenotypes. Figure S4. Amino acid sequences’ alignment between WT and *cde6*-related mutants; Figure S5. The results of RiceXPro predicting the expression pattern of the *CDE6*; Figure S6. Amino acid sequences’ alignment between *hfp108* and *cde6* mutants.
