# Supplementary material for: CDE6 Regulates Chloroplast Ultrastructure and Affects the Sensitivity of Rice to High Temperature
Source: Plants (Basel). 2026 Jan 17;15(2):284. doi: 10.3390/plants15020284 (PMC12845262; doi:10.3390/plants15020284)
Supplement: Supplementary file 1 [file plants-15-00284-s001.zip › plants-4043108-supplementary.pdf]

Supplementary Material

Supplementary Data S1. All primer sequences used in this study

Purpose	Primer name	Sequence
Mutmap+ mutant SNP detection	CDE6-F	TCGGACAAGAGGCAAGCTAC
	CDE6-R	ACCAGCCCAGACATAGGTTC
Knockout experiment	U3F1	GTTACTAGATCGGGCCCAGGAATCTTTAAACATACGAACAGATC ACT
	CDE6KD-R1	GCGGGGGAGACGGCGTGGAGTGCACCAGCCGGGAATCGAAC
	CDE6KD-F1	GCACTCCACGCCGTCTCCCCCGCGTTTTAGAGCTAGAAATAGCA AGTTAAAATAAG
	gRNAR1	TTCCATCCACTCCAAGCTCTTGAA
	U6F2	AGCTTGGAGTGGATGGAATTTTTTCCTGTAGTTTTCCCACAACC
	CDE6KD-R2	TCTTCAGCTAATTTAGCAAC TGCACCAGCCGGGAATCGAAC
	CDE6KD-F2	GCAGTTGCTAAATTAGCTGAAGAGTTTTAGAGCTAGAAATAGCA AGTTAAAATAAG
	gRNAR2	AGCTTGCATGCCTGCAGGGTAAAACGGAGGAAAATTCCATC
RT-qPCR	NYC4-RT-F	GGTTATCCTGCTGCTTGCTGG
	NYC4-RT-R	AGATGTTCTCGCCGTACTTCCC
	NYC3-RT-F	GGACAAGGAGGAGAATGACAGG
	NYC3-RT-R	GAAGGCAACCATCAGGTGAGTAG
	NYC1-RT-F	GAAGGACATCGTGGATGGCTAC
	NYC1-RT-R	TTGGACATTTCTGCGGCTCT
	DVR-RT-F	CCTACCTCATCGCCAACCC
	DVR-RT-R	CCGACAGCAACCAAACCACA
	SHA1-F	TTCTCGTCGATGACCTCCTG
	SHA1-R	AGGGTGGTTGACGCATCTTA
	SHA2-F	GTCGGTCTGTGTTGCGATTTG
	SHA2-R	CCTCCATTTCCCACACAGCTT
	WLP2-F	AAGGCTGTGGCTTGATTGACA
	WLP2-R	TTAGGCCCAATTTTGCTATTTTG
	FtsZ-F	TGACCAACTACAACGTGGTCCC
	FtsZ-R	GCCAGTGTATGTTTTGCCGAAG
	RPOA-F	AGGTGCCACAAGGAAAGATCTGGT
	RPOA-R	TCAGCAGGGCTTTGTCACTAGGAA
	RPOB-F	TGGGAAGATGCCACAAGGAGAGAT
	RPOB-R	TCCGCAGCATATTTCTCCACCAGT

	PsaB-F	TCCGCCGTATAAACTTGATGCCCT
	PsaB-R	TGGGTTGCCGTTGTTGTATGCTTC
	PsbA-F	ATCTGGATGGGTGTGGCTAGCTTT
	PsbA-R	AGTACGCATGCTCCCAGACATCAA
	CHLI-F	CACCGTGGAGAGAGAGGGTA
	CHLI-R	ACAGCACCAAGGTTACTCCG
	OsHSFA2a-F	GCGTCCAGGAGAGTAACAGC
	OsHSFA2a-R	GTCATCCTCCTCGTCGTTGT
	OsHSFA2b-F	GGGCTTGTTGGTGAGGAGAAC
	OsHSFA2b-R	CATCTTCTCCGACAACACATTCA
	OsHSP101-F	GGAGGAGTACCGCAAGTACG
	OsHSP101-R	GAGGTCGATCGCTTTGTCAG
	OsHSP71.1-F	GAGGCCTATCTCGGAAGCAC
	OsHSP71.1-R	GTGAGGAGGGAGACATCGAA
Subcellular localization	LBL5-GFP-F	GGGTACCCGGGGATCCTCTAGAATGCCGCCGCTCCAC
	LBL5-GFP-R	CTTGCTCACCATGGTACTAGTAATTTTCATGAGTTCCTGTTCC

Supplementary Data S2. Segregation ratio of hybrid progeny

Hybridization combinations	Wild-type	Mutant	Separation ratio	$\chi^2_{(3,1)}$($\chi^2_{0.05,1}$=3.84)
cde6×WT	443	140	2.82:1	0.302

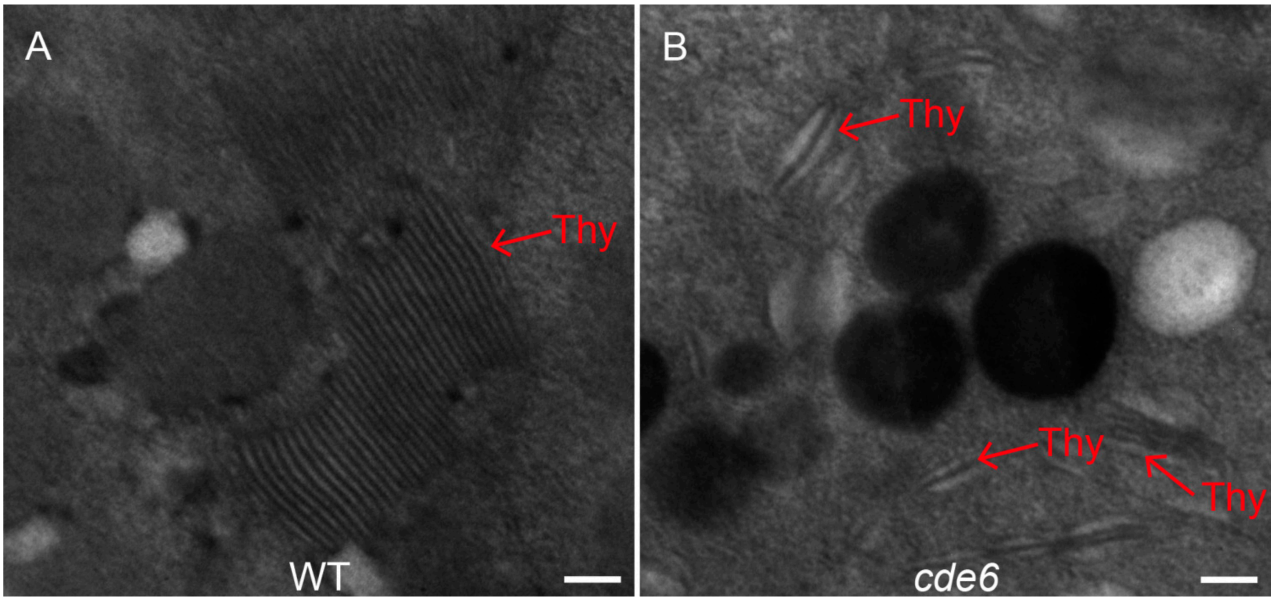

Figure S1. Chloroplast ultrastructures of wild-type and *cde6* mutant rice. (A) Chloroplast ultrastructure of wild-type (WT) rice; (B) Chloroplast ultrastructure of cde6 mutant rice. Scale bars = 100 nm.

```
                                                                    Section 1
       (1) 1       10        20        30        40             55
    WT (1) MPPLHAVSPAAAAAPPRALSSAARVPQRPGCVPERPNILSSSTNFMSLRAGPMRF
  cde6 (1) MPPLHAVSPAAAAAPPRALSSAARVPQRPGCVPERPNILSSSTNFMSLRAGPMRF
                                                                    Section 2
      (56) 56            70        80        90        100       110
   WT (56) YSRPLILQNSDKRAVLRHATIEEIEAEKSVIEDQARERMEKAIETVQNNFNTVRT
 cde6 (56) YSRPLILQNSDKRAVLRHATIEEIEAEKSVIEDQARERMEKAIETDQNNFNTVRT
                                                                    Section 3
     (111) 111      120       130       140       150            165
  WT (111) GRANPAMLDRIEVEYYGTPVNLKSIAQINTPDATSLLIQPYDKSSLKLIEKTIVA
cde6 (111) GRANPAMLDRIEVEYYGTPVNLKSIAQINTPDATSLLIQPYDKSSLKLIEKTIVA
                                                                    Section 4
     (166) 166           180       190       200       210       220
  WT (166) ANLGVTPSNDGEVIRVTVPPLTSDRRKELAKTVAKLAEEGKVAIRNIRRDAIKAY
cde6 (166) ANLGVTPSNDGEVIRVTVPPLTSDRRKELAKTVAKLAEEGKVAIRNIRRDAIKAY
                                                                    Section 5
     (221) 221      230       240       250            266
  WT (221) DKLEKEKKLSEDNVKDLSADLQKVTDEYMKKIEAIQKQKEQELMKI
cde6 (221) DKLEKEKKLSEDNVKDLSADLQKVTDEYMKKIEAIQKQKEQELMKI
```

Figure S2. Amino acid sequences alignment between WT and *cde6* mutants. The amino acid sequences were aligned using Vector NTI.

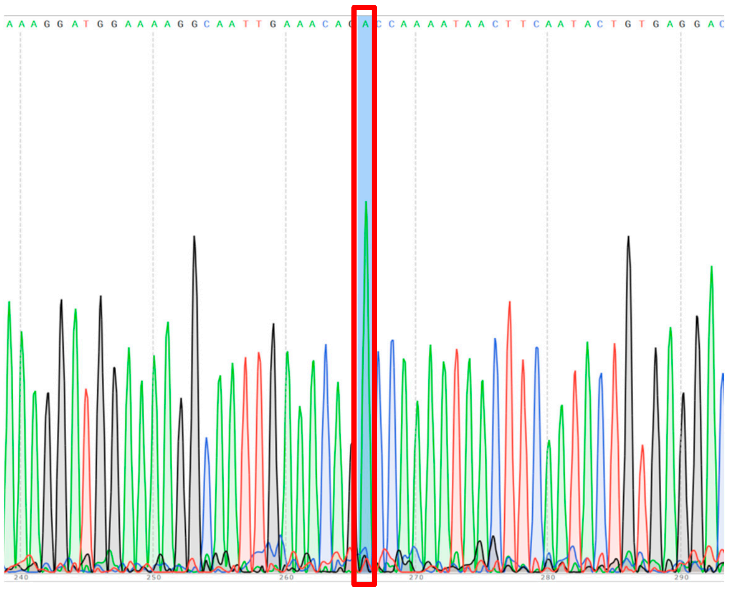

Figure S3. Cosegregation verification of genotypes and phenotypes.

```
                                                              Section 1
      (1) 1         10             20             30             40                 55
WT    (1) MPPLHAVSPAAAAAPPRALSSAARVPQRPGCVPERPNILSSSTNFMSLRAGPMRF
cp-1  (1) MPPLHAVSPAAAAAPPRALSSAARVPQRPGCVPERPNILSSSTNFMSLRAGPMRF
cp-2  (1) MPPLHAVSPAAAAAPPRALSSAARVPQRPGCVPERPNILSSSTNFMSLRAGPMRF
                                                              Section 2
     (56) 56            70             80             90             100            110
WT   (56) YSRPLILQNSDKRAVLRHATIEEIEAEKSVIEDQARERMEKAIETVQNNFNTVRT
cp-1 (56) YSRPLILQNSDKRAVLRHATIEEIEAEKSVIEDQARERMEKAIETVQNNFNTVRT
cp-2 (56) YSRPLILQNSDKRAVLRHATIEEIEAEKSVIEDQARERMEKAIETVQNNFNTVRT
                                                              Section 3
    (111) 111       120            130            140            150            165
WT  (111) GRANPAMLDRIEVEYYGTPVNLKSIAQINTPDATSLLIQPYDKSSLKLIEKTIVA
cp-1(111) GRANPAMLDRIEVEYYGTPVNLKSIAQINTPDATSLLIQPYDKSSLKLIEKTIVA
cp-2(111) GRANPAMLDRIEVEYYGTPVNLKSIAQINTPDATSLLIQPYDKSSLKLIEKTIVA
                                                              Section 4
    (166) 166           180            190            200            210            220
WT  (166) ANLGVTPSNDGEVIRVTVPPLTSDRRKELAKTVAKLAEEGKVAIRNIRRDAIKAY
cp-1(166) ANLGVTPSNDGEVIRVTVPPLTSDRRKELAKTVAKLAERRQGCYKEHKKRCHQSL
cp-2(166) ANLGVTPSNDGEVIRVTVPPLTSDRRKELAKTVAKLAEKARLL-----------
                                                              Section 5
    (221) 221       230            240            250                267
WT  (221) DKLEKEKKLSEDNVKDLSADLQKVTDEYMKKIEAIQKQKEQELMKI-
cp-1(221) R----------------------------------------------
cp-2(209) -----------------------------------------------
```

Figure S4. Amino acid sequences alignment between WT and *cde6*-related mutants. Comparison of CDE6 amino acid sequences among the WT, and two knock out mutants (*cr-1*, *cr-2*). The amino acid sequences were aligned using Vector NTI.

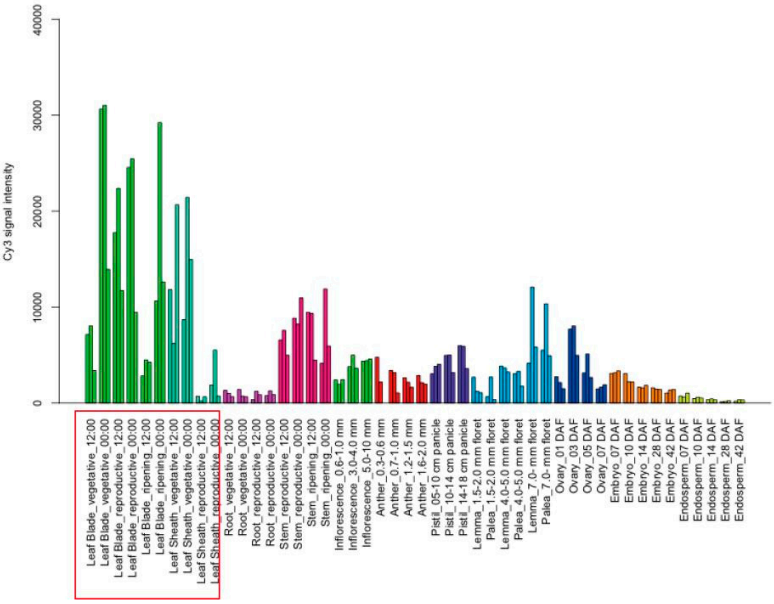

Figure S5. The results of RiceXPro predicting the expression pattern of the *CDE6*.

```
                                                                        Section 1
              (1)  1         10        20        30        40             55
       hfp108 (1)  MAASFSSTAPTTPVLRFRANYSKPLLSLPDSCLRIISSAISPSTRLIACSFKTDK
       cde6   (1)  -MPPLHAVSPAAAAAPPRALSSAARVPQRPGCVPERPNILSSSTNFMSLRAG---
                                                                        Section 2
             (56)  56            70        80        90        100      110
       hfp108 (56) LPLGAGVNLSGGPVVKRSLQKRLVIRSATIEEIEAEKSAIETDVKSKMEKTIETL
       cde6   (52) -----PMRFYSRPLILQNSDKRAVLRHATIEEIEAEKSVIEDQARERMEKAIETV
                                                                        Section 3
            (111)  111       120       130       140       150          165
       hfp108 (111) RTSFNSIRTGRSNAAMLDKIEVEYYGSPVSLKSIAQISTPDGSSLLLQPYDKSSL
       cde6   (102) QNNFNTVRTGRANPAMLDRIEVEYYGTPVNLKSIAQINTPDATSLLIQPYDKSSL
                                                                        Section 4
            (166)  166           180       190       200       210      220
       hfp108 (166) KAIEKAIVNSDLGVTPNNDGDVIRLSLPPLTSDRRKELSKVVAKQSEEGKVALRN
       cde6   (157) KLIEKTIVAANLGVTPSNDGEVIRVTVPPLTSDRRKELAKTVAKLAEEGKVAIRN
                                                                        Section 5
            (221)  221       230       240       250       260          275
       hfp108 (221) IRRDALKSYDKLEKEKKLSEDNVKDLSSDLQKLIDVYMKKIEELYKQKEKELMKV
       cde6   (212) IRRDAIKAYDKLEKEKKLSEDNVKDLSADLQKVTDEYMKKIEAIQKQKEQELMKI
```

Figure S6. Amino acid sequences alignment between *hfp108* and *cde6* mutants. Comparison of CDE6 amino acid sequences between the *hfp108* and the *cde6* mutant. The amino acid sequences were aligned using Vector NTI.
